# How Tobacco Smoking Injures Yale Students

**Published:** 1891-08

**Authors:** 


### HOW TOBACCO SMOKING INJURES YALE STUDENTS.

Dr. Seaver, of Yale College, is waging war upon the habit of tobacco smoking, which some of the students there indulge in. He is the physician of the college and the professor of athletics, a man of science who follows scientific methods in any investigation he may undertake. He has been engaged for eight years in observing the effects of tobacco smoking upon the minds and bodies of Yale students, and he has just published a remarkable budget of statistics. Dr. Seaver informs the public that the students of Yale who indulge in tobacco smoking are inferior in physical vigor and mental ability to those that do not. According to his reckoning the smokers have less lung power than the anti-smokers ; they have less chest-inflating capacity ; they are of less bodily weight and they are even of less height. The muscular and nervous power of the smoking students is notably and noticeably less than that of the anti-smoking. From an athletic point of view, therefore, the Yale professor of athletics considers himself justified in waging war upon the tobacco habit. Not only in a physical way, but also in an intellectual way, the Yale smokers are inferior to the anti-smokers. The smoking habit is disadvantageous to scholarship. Of those students who, within a given time, have received junior appointments above dissertations, only 5 per cent. were smokers, and very few smokers received appointments of any kind. It would seem, therefore, that the brain power and the scholarship of the smokers at Yale are far inferior to those of the anti-smokers. The demonstrations of Dr. Seaver appear to be influencing the Yale mind. He is able to report that 70 per cent. of the senior class do not smoke, and the leading athletes do not smoke, and that not a single candidate for the rowing crew is a smoker. Young America, athletic, intellectual, and ethical, can ruminate upon the Yale statistics collected by Dr. Seaver.

